# L-cysteine/MoS_2_ modified robust surface plasmon resonance optical fiber sensor for sensing of Ferritin and IgG

**DOI:** 10.1038/s41598-023-31152-3

**Published:** 2023-03-31

**Authors:** Priyanka Thawany, Ashima Khanna, Umesh K. Tiwari, Akash Deep

**Affiliations:** 1 Academy of Scientific and Innovative Research (AcSIR), Ghaziabad, Uttar Pradesh 201002 India; 2 CSIR-Central Scientific Instruments Organization (CSIR-CSIO), Sector 30C, Chandigarh, 160030 India; 3 Present Address: Institute of Nano Science and Technology, Sector-81, S.A.S. Nagar, Punjab India

**Keywords:** Biotechnology, Biomarkers, Optics and photonics

## Abstract

L-cysteine conjugated molybdenum disulphide (MoS_2_) nanosheets have been covalently attached to a gold coated surface plasmon resonance (SPR) optical fiber to prepare a robust and stable sensor. Owing to the multifunctionality of the deposited nanosheet conjugate, the antibodies are also covalently conjugated in the subsequent step to realize the design of a SPR optical fiber biosensor for the two important bioanalytes namely, Ferritin and Immunoglobin G (IgG). The different stages of the biosensor preparation have been characterized and verified with microscopic and spectroscopic techniques. A uniform and stable deposition of the L-cysteine/MoS_2_ nanosheets has allowed the biosensor to be reused for multiple times. Unlike the peeling-off of the MoS_2_ coatings from the gold layer reported previously in the case of physically adsorbed nanomaterial, the herein adopted strategy addresses this critical concern. It has also been possible to use the single SPR fiber for both Ferritin and IgG bioassay experiments by regenerating the sensor and immobilizing two different antibodies in separate steps. For ferritin, the biosensor has delivered a linear sensor response (SPR wavelength shifts) in the concentration range of 50–400 ng/mL, while IgG has been successfully sensed from 50 to 250 µg/mL. The limit of detection for Ferritin and IgG analysis have been estimated to be 12 ng/mL and 7.2 µg/mL, respectively. The biosensors have also been verified for their specificity for the targeted molecule only. A uniform and stable deposition of the nanomaterial conjugate, reproducibility, regeneration capacity, a good sensitivity, and the specificity can be highlighted as some of key features of the L-cysteine/MoS_2_ optical fiber biosensor. The system can be advocated as a useful biosensor setup for the sensitive biosensing of Ferritin and IgG.

## Introduction

Portable diagnostics technologies or point-of-care (POC) devices are the need of the hour to cater the demands of affordable healthcare. The clinical significance of the routine detection of Ferritin and Immunoglobins is increasing for which it becomes of utmost importance to develop rapid sensing techniques^1–3^. Ferritin is an iron containing protein in blood and its levels help in estimating the amount of iron stored in the body^4^. The normal levels of Ferritin in human may vary from 10 to 340 µg/L^5^. A lower-than-normal concentration of ferritin in an indicator of low iron content in the body (i.e., anaemic condition) whereas a higher-than-normal level points out an excess storage of iron which is also a health disorder^6^. The elevated concentrations of Ferritin could be associated with inflammatory conditions, hyperthyroidism, rheumatoid arthritis, liver diseases and even certain forms of cancer^7^. Immunoglobulin gamma (IgG) is formed by the short chain of amino acids, connected through peptide bonds^8^. The levels of IgG are indicative of a person’s immune status. The immunoglobulin test is done to diagnose the immunodeficiencies^9^. An increased level of IgG in blood is associated with disorders like hyperthyroidism and systemic lupus erythematosus. The detection of IgG is also important to test the presence of novel coronavirus in human body as well as to assess the efficacy of vaccines during the course of immunization^10,11^. The normal range of IgG in human blood range from 6 to 16 g/L. In particular, the detection of IgG in both low and high concentration range assumes a clinical significance^12^.

To overcome the limitations (e.g., large processing time, multiple step processing, etc.) of the enzyme-linked immunosorbent assay (ELISA) technique, many biosensing methods have been developed over the past decade for the detection of Ferritin and IgG^2,7,13–18^. These biosensors are based on electrochemical, optical, piezoelectrical, and other such techniques. In recent years, the optical fiber based surface plasmon resonance (SPR) sensing platforms have also emerged as promising devices for the detection of biomolecules^19–24^. A relatively low-cost fabrication, absence of electromagnetic interference, flexibility, portability, and rapid processing time have facilitated the development of optical fiber biosensors in wide variety of applications, including pathogens detection, food safety, disease marker detection to name a few^23,24^.

The fabrication of an optical fiber SPR sensor involves the uncladding of a part of the fiber followed by the deposition of a thin film of a noble metal (e.g., gold, silver) to excite the collective electron oscillation (plasmons) at the metal–dielectric interface. The resonance frequency depends on factors like type of metal layer, doping of fiber, refractive index (RI) of the surrounding medium, and receptor layer^25^. An evanescent wave is produced at the metal–dielectric interface, which decays exponentially in both the medium making the dielectric medium sensitive to the RI changes upto a few nanometers. The deeper the penetration of the evanescent wave, better is the sensitivity, meaning that the interaction of the biorecognition element with the analytes can be monitored with greater precision^26,27^.

In the recent past, the development of SPR optical fiber (SPR-OF) sensors for human IgG have been reported from some research groups. For instance, polydopamine (PDA)-accelerated deposition of gold film was used to fabricate an SPR-OF sensor^27^. This sensor was further immobilized with specific antibodies and the resulted system could deliver the detection of human IgG from 0.5 to 40 μg/mL. A report described the use of an MMF–NCF–MMF structure for developing the sensor for human IgG^28^. The sensor was constructed with a noncore fiber (NCF) sandwiched between two multimode fibers (MMF). A gold film of 50 nm thickness was sputtered, and the goat anti-human IgG-human IgG was applied as a bioconjugated pair. A D-type OF-SPR biosensor modified with poly dimethyl diallyl ammonium chloride and Poly(sodium-p-styrenesulfonate) layers was also reported for the detection of human IgG^29^. Based on the application of HIgG and goat anti-HIgG bioconjugate pair, the sensitivity of the above sensor toward IgG was reported to be 91 nm/(mg/mL). As such, the SPR-OF sensors for Ferritin have not been investigated to the best of the authors’ knowledge.

The fiber optics setups for SPR still face a challenge in terms of their sensitivity compared to the classic prism configuration which has made its way to the commercial market for bioanalysis. Many efforts have been made to increase the penetration depth by adopting strategies like tapering, bending of the fiber, changing the angle of incidence, and wavelength of light^30,31^. Nonetheless, the structural changes in fiber make them prone to breakage and as a result a precise handling is required^32^. The layers of 2-dimenstional (2D) nanomaterials, such as graphene and molybdenum disulphide (MoS_2_), over the optical fiber SPR sensors have been introduced to improve the characteristics of such sensors^33,34^. These 2D nanomaterials are known for their high electron mobility, anisotropic electron transport behavior, and large specific surface areas. Their layering on SPR optical fiber sensors increases the penetration depth of the evanescent wave thereby facilitating a better interaction and this in turn improves the sensitivity of sensors allowing a precise detection of the trace biomolecules. In one of the recent studies, Kaushik et al. applied a MoS_2_ layer over the optical fiber SPR sensor and then immobilized antibodies to target the detection of *E. coli*^35^. Later, Hong Song et al. observed that the coating of MoS_2_ over fiber surfaces was not necessarily uniform and it was also prone to fallout^36^. To address the issue, they suggested the use of a U-bend fiber with a MoS_2_ layer sandwiched with the gold layers. The above researchers used PDA to conjugate the antibodies. However, the use of a fiber with small radius comprised on the surface area, which is not ideal for optimal interactions.

A simple physical absorption of MoS_2_ nanosheets over the optical fiber SPR sensor surface lacks the robustness of coating. Also, a simple adsorption of antibodies also does not guarantee the realization of a sensitive and stable sensor system. In the present research work, for the first time, we describe the use of a L-Cysteine modified MoS_2_ nanosheets to design a sensitive, stable, and precise optical fiber SPR immunosensor for Ferritin and IgG. The multifunctional nature of the L-Cysteine/MoS_2_ nanostructure ensures adherence of the MoS_2_ over the gold later via disulphide linkage, while the pendant carboxyl groups have been utilized for oriented grafting of the antibodies via covalent linkage. As a result, a uniform and stable coating of MoS_2_ is achieved and the resulting biosensor has allowed the detection of the selected proteins over a wide concentration range, which is of clear clinical significance.

## Experimental

### Materials

The silicon core polymer clad optical fiber (400 µm, multimode) was purchased from Thorlabs. Ferritin monoclonal antibodies were purchased from Thermo Fisher, India. IgG from human serum (salt-free, lyophilized powder), Ferritin (equine spleen), and Rabbit Anti-Human IgG polyclonal antibodies were purchased from Sigma. All other chemicals including Molybdenum disulphide (MoS_2_, < 2 µm) were high purity grade materials from Sigma/Merck.

### Characterizations

The materials and surfaces were characterized for their different features and properties on instruments like UV–Vis spectrophotometer (Varian Cary 4000), Fourier Transform Infrared spectrophotometer (FTIR, Nicolet iS10), Raman spectrometer (Invia, Renishaw, 785 nm laser), X-ray diffractometer (XRD, D8 Advance, Bruker), Field Emission Scanning Electron Microscope (FESEM, Hitachi, SU8010), and High Resolution Transmission Electron Microscope (HRTEM, JEOL, JEM 2100 Plus). The sensing setup was formed with a spectrometer (ULS2048XL-EVO, Avantes), and a light source (Ocean Optics, HL-2000).

### Synthesis of MoS_2_ nanosheets

The MoS_2_ nanosheets were synthesized by a liquid phase exfoliation method^37^. Briefly, 1 g of MoS_2_ powder was first left in contact with liquid nitrogen for 6 h. Then, the powder was mixed with 500 mL of a mixture of isopropyl alcohol (IPA) and water (1:1, v/v). Next, 4 g of sodium borohydride (NaBH_4_) was added, and the mixture was sonicated in an ultrasonic bath for 5 h. It was followed by a further probe sonication step for 6 h. In the process, the probe tip was set for 7 s on and 5 s off steps (amplitude = 98%, frequency = 20 kHz). The reaction contents were then centrifuged at 8000 rpm for 30 min to collect the supernatant, which contained water-soluble defect-rich MoS_2_ nanosheets.

### Preparation of L-cysteine conjugated MoS_2_ nanosheets

The MoS_2_ nanosheet solution was heated at 60 degrees for overnight. Next, 100 uL of 10 mM L-cysteine solution was added and the mixture was incubated under stirring conditions for 2 h to prepare the L-Cysteine conjugated MoS_2_ nanosheets.

### Fabrication of L-cysteine/MoS_2_ modified optical fiber SPR biosensor

The jacket (polymer cladding of 25 µm) of the 400 µm multi-mode optical fiber was removed with a razor blade to expose the fiber. It was then etched with hydrofluoric acid for 40 min (etching rate 3 µm/min). This step provided a surface roughness for facilitating the sputtering of the gold layer. The roughness of the fiber also helps in a better coupling of plasmon^38^. The gold layer of around 50 nm thickness was deposited onto the etched optical fiber using a Magnetron sputtering unit (Excel Instruments, India).

The gold coated optical fiber was cleaned with methanol and deionized water. It was then left in contact with in 400 µL of L-Cysteine/MoS_2_ solution for 30 min, followed by drying under a halogen lamp for 5 min. Based on the preliminary SPR response curves, 4 numbers of coating cycles were optimized to obtain a stable signal from the OF-SPR sensor. The coating of L-Cysteine/MoS_2_ layer over the optical fiber was fairly stable. We did not face any fall-out/peeling issue of film from the fiber during the experiments.

For the immobilization of antibodies, the above prepared sensor was incubated with a mixture of 500 µL of 0.1 mM EDC + 2 mM NHS for 30 min. Next, 500 µL of 10 µg/mL antibody (anti-IgG and anti-Ferritin antibodies in separate set of experiments) were introduced and left to incubate for 30 min. The above step resulted in a covalent attachment of the respective biorecognition elements to form the biosensor for Ferritin or IgG. After washing with 400 µL of PBS solution, the non-specific binding sites were blocked by flushing the optical fiber biosensor with 1% BSA solution in PBS (pH 7.4). Post antibody immobilization, the sensor can be stored under refrigerated conditions (e.g., 4 °C) to ensure its prolonged shelf-life.

### Experimental setup

**Figure 1:**
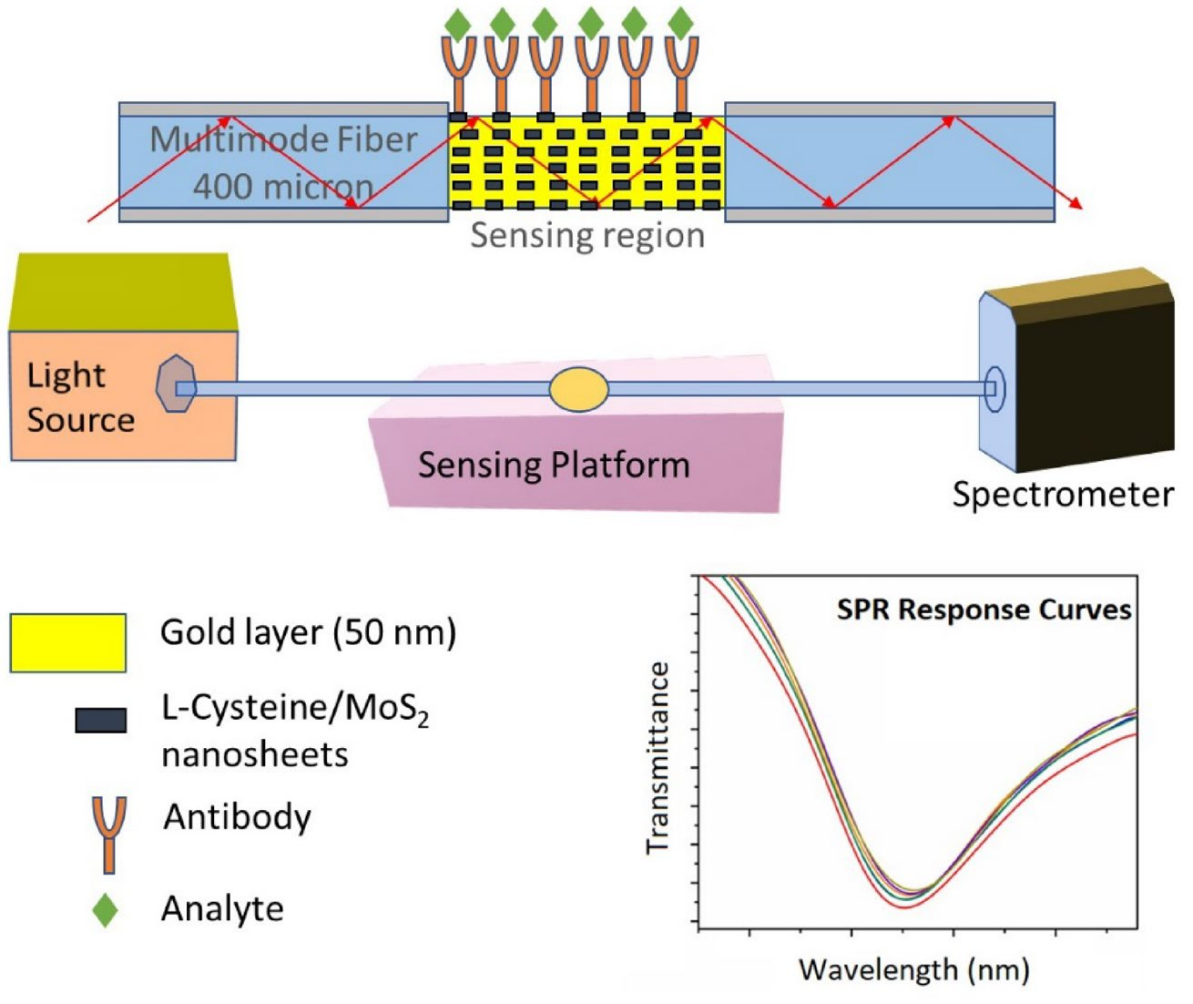
Experimental setup of the MoS_2_ modified OF-SPR biosensor.

The prepared optical fiber biosensor, cleaved at both ends, was connected in line with the help of a bare fiber terminator (Thor labs) to the spectrometer and the Halogen light source. A Teflon flow cell was used to introduce the solutions and carry out different modifications, functionalizations, and bioassays. The scheme of the experimental setup is depicted in Fig. 1.

## Results and discussion

### Characterization of the synthesized MoS_2_ nanosheets

**Figure 2:**
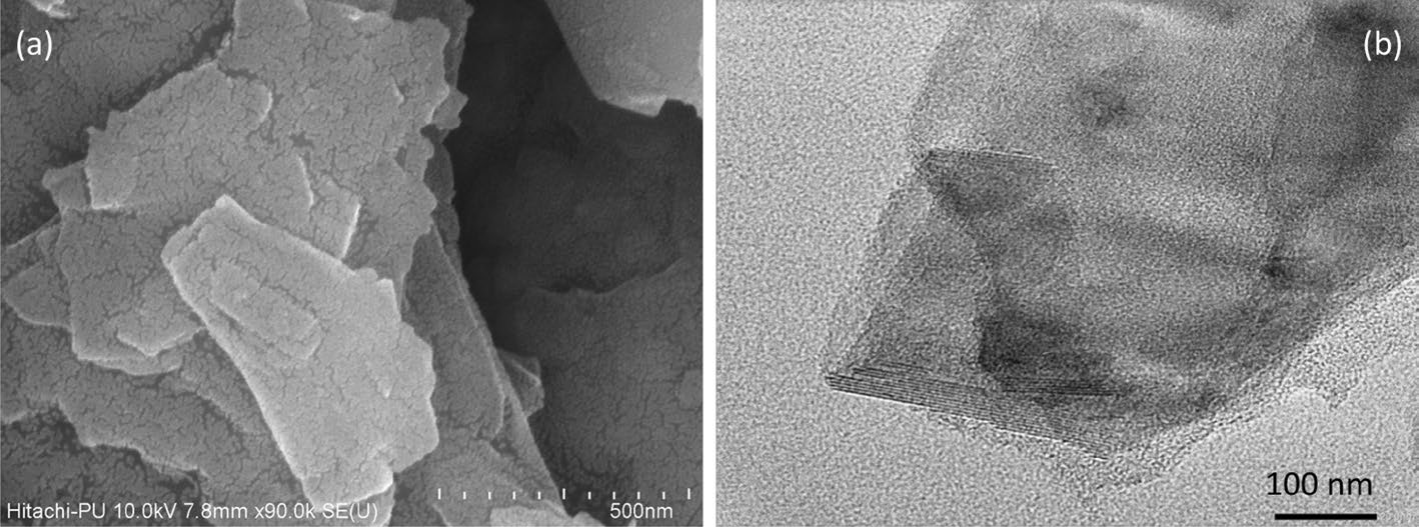
(**a**): FE-SEM; (**b**) HR-TEM image of synthesized MoS_2_ nanosheets.

Figure 2a and b shows the FESEM and HRTEM images of the synthesized L-Cysteine modified MoS_2_ nanosheets. The FESEM investigations highlights the preparation of uniform nanosheets with lateral dimension in the range of 100–400 nm. The HRTEM studies have revealed the formation of few-layered (~ 10) MoS_2_ nanosheets thereby suggesting their thickness to be around 6.5 nm.

**Figure 3:**
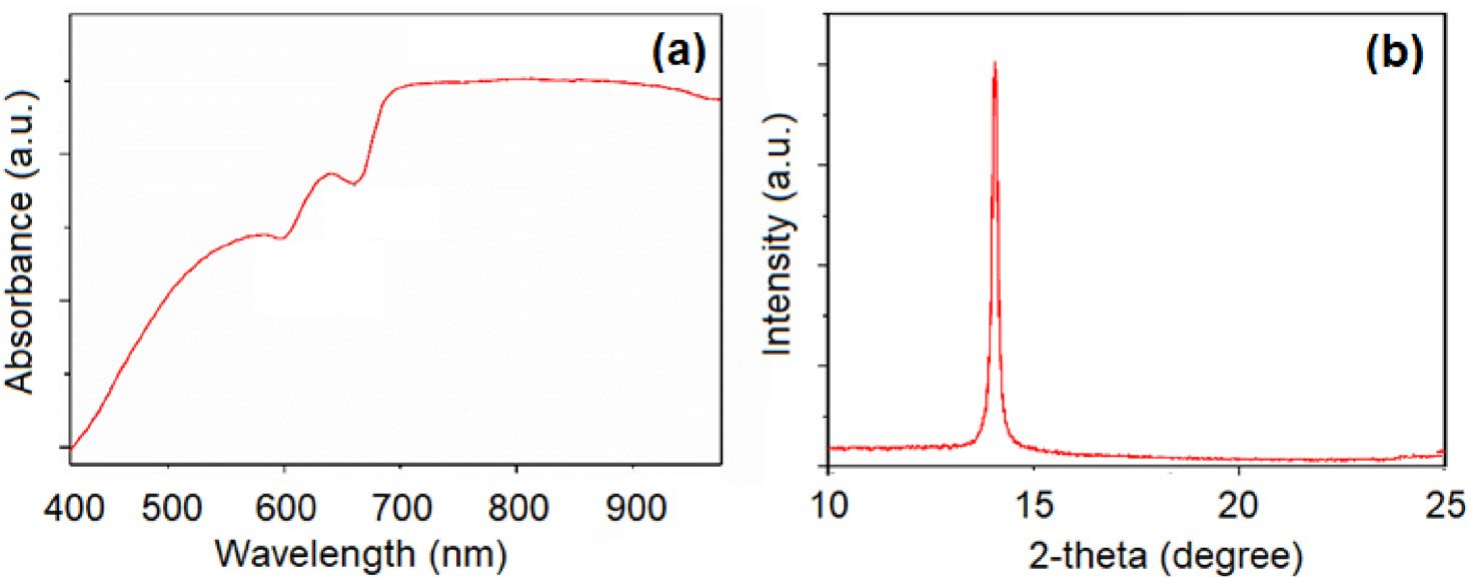
(**a**): Absorption spectrum of the synthesized MoS_2_ nanosheets; (**b**) XRD pattern of MoS_2_ nanosheets film on a silicon substrate.

Different evidences have been collected to verify the successful preparation of the MoS_2_ nanosheets and their subsequent modification with L-Cysteine. Figure 3a shows the UV–Vis spectrum of the synthesized MoS_2_ nanosheets. The well-resolved peaks in the region of 580–700 nm account for the successful formation of the MoS_2_ nanosheets. The absorption bands can be referred to the B-excitonic (585 nm) and A-excitonic (640 nm) characters^39^. The recorded XRD pattern of the MoS_2_ nanosheets is shown in Fig. 3b. A major diffraction peak, centered at 14.5°, correspond to the (002) plane, which is a critical supporting indicator to confirm the formation of the MoS_2_ nanosheets^39^.

**Figure 4:**
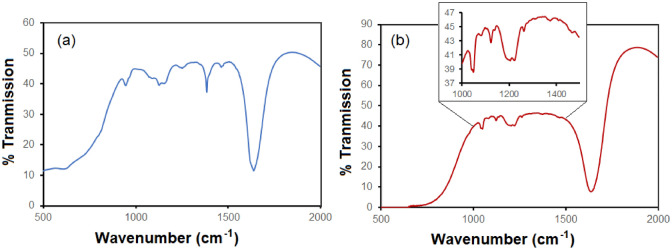
(**a**): FTIR spectrum (Transmission mode) of synthesized MoS_2_ nanosheets; (**b**): FTIR (Attenuated Total Reflection mode) spectrum of antibody conjugated L-Cysteine/MoS_2_ nanosheets deposited on a silicon substrate.

Figure 4 present the FTIR spectra of the MoS_2_ nanosheets and antibody (anti-Ferritin) conjugated L-Cysteine/MoS_2_ nanosheets. It may be noted here that the FTIR spectrum of the antibody conjugated L-Cysteine/MoS_2_ nanosheets (thin film deposited on a silica surface) was recorded in the Attenuated Reflection mode. The FTIR spectrum of the MoS_2_ nanosheets contains bands at 485, 615, 946, 1130–1160, 1384, and 1640 cm^−1^. The bands at 485 and 615 cm^−1^ are associated with the Mo–S bonds. A band at 946 cm^−1^ is associated to the S–S bonds. The hydroxyl group and Mo–O bonds along with their stretching vibrations are referred from the absorption bands at 1130–1160 and 1640 cm^−1^^40^. After antibody conjugation, a new band of Amide III (C–N stretching) appears at 1220–1230 cm^−1^ (Fig. 4b)^41^. Other bands related to the MoS_2_ structure are also present in the antibody conjugated L-Cysteine/MoS_2_ sample. Therefore, the FTIR studies have provided a useful information about the successful immobilization of the antibodies on the L-Cysteine/MoS_2_ nanosheets.

**Figure 5:**
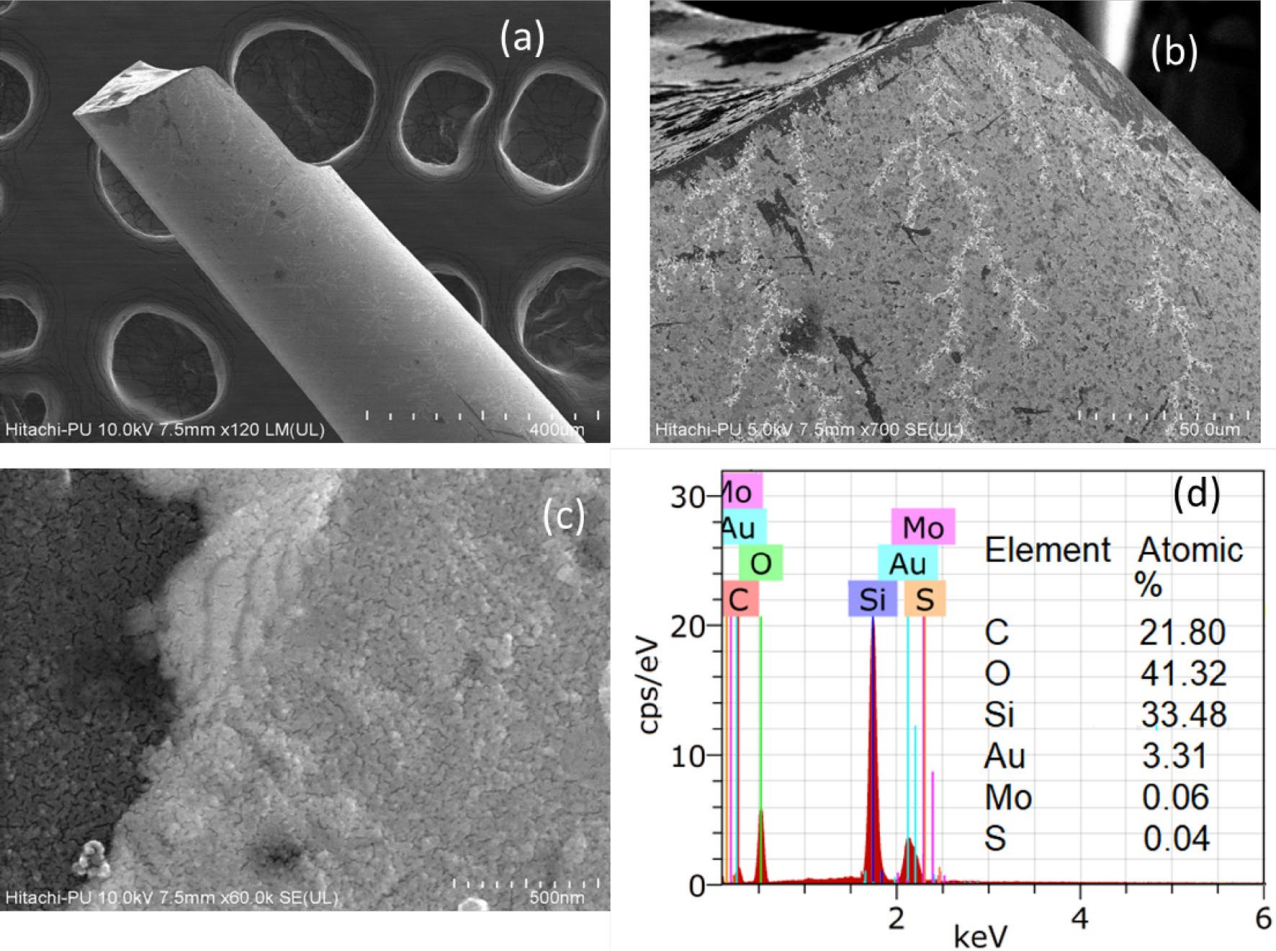
(**a**): FESEM image of a gold coated optical fiber; (**b**,**c**): FESEM images of L-Cysteine/MoS_2_ deposited gold layered optical fiber; (**d**): EDX based elemental ratio analysis of L-Cysteine/MoS_2_ deposited gold layered optical fiber.

Figure 5a shows the SEM image of a gold coated optical fiber. The fiber was broken for these studies for a better analysis and easy mounting of the samples. A uniform gold coating on the fiber is well indicated from the SEM investigations. The SEM images with different magnifications of the L-Cysteine/MoS_2_ modified optical fiber are shown in Fig. 5b, c. These studies have provided useful evidence about the successful uniform modification of the gold coated optical fiber with L-Cysteine/MoS_2_ nanosheets. An interface between gold layer and MoS_2_ nanosheets is also well highlighted in Fig. 5c. Figure 5d mentions the EDX based elemental assessment of the elemental ratios. The presence of Si is from the silicon wafer on which the sample was mounted. Though the elemental ratio of Mo seems low as several factors can be considered in the EDX analysis, nonetheless this study, in conjunction with SEM imaging, has provided a strong indication about the uniform modification of the gold coated optical fiber with the L-Cysteine/MoS_2_ nanosheets.

### Response studies of the Ab/L-cysteine/MoS_2_/OF-SPR biosensor toward Ferritin and IgG

The significance of the portable bioassays for Ferritin and IgG have been explained in the Introduction section. The benefits of using L-Cysteine conjugated MoS_2_ lie in the multifunctional nature of the developed interface. L-Cysteine has helped in a better and uniform attachment of MoS_2_ nanosheets over the gold coated OF. Therefore, one can expect a greater and uniform loading of the antibodies also. A robust attachment of the L-Cysteine/MoS_2_ over the OF surface allowed us to reuse the same fiber for multiple biosensing studies (e.g., 5–7 times) without requiring the need of the remodification of the fiber with the nanomaterial conjugate. We observed no change in the basic baseline (SPR wavelength) response.

The response of the L-Cysteine/MoS_2_/OF-SPR sensor (i.e., without antibody) was first recorded to rule out any physical interaction between the material layer and the analyte (Ferritin and IgG). The SPR responses were recorded for the L-Cysteine/MoS_2_/OF-SPR sensor for dilute analyte (100 ng/mL Ferritin and 50 µg/mL IgG) solutions. Since there was no shift observed in the SPR curves, it ruled out any interaction between the L-Cysteine/MoS_2_ and the analytes.

For measuring the SPR response of the biosensor, firstly a reference reading was recorded by introducing 400 µL of PBS in the Teflon flow cell. Based on the preliminary investigations, the above volume was optimized to attain a maximum transmission value. The larger volumes did not affect the results. Therefore, it can be mentioned that only a small sample volume is required for attaining the response from the Ab/L-Cysteine/MoS_2_/OF-SPR biosensor, which is a critical advantage for clinical applications.

The response time of the Ab/L-Cysteine/MoS_2_/OF-SPR biosensor toward both Ferritin and IgG was also optimized. An incubation time of 4 min was sufficient to obtain a stable SPR response from the spectrometer for different concentrations of both analytes. Therefore, it can be highlighted that the biosensor has a rapid response time. All further SPR response measurements were taken after 4 min of incubation between the analyte and the Ab/L-Cysteine/MoS_2_/OF-SPR biosensor.

In a typical bioassay experiment, the biosensor was left in contact with 400 µL of analyte solution. After 4 min, as the antigen–antibody interaction was complete, the analyte solution was removed and a fresh 400 µL aliquot of PBS was introduced. This step allowed to nullify other factors than the antigen–antibody complex formation and provided a much accurate assessment of the SPR wavelength shift with respect to the refractive index changes. This strategy ensured that the SPR response (i.e., wavelength shift) was not from the RI changes of the analyte solution but from the desired antigen–antibody complex formation. This experimental strategy was followed for all the bioassay measurements for Ferritin and IgG.

#### Ferritin detection

**Figure 6:**
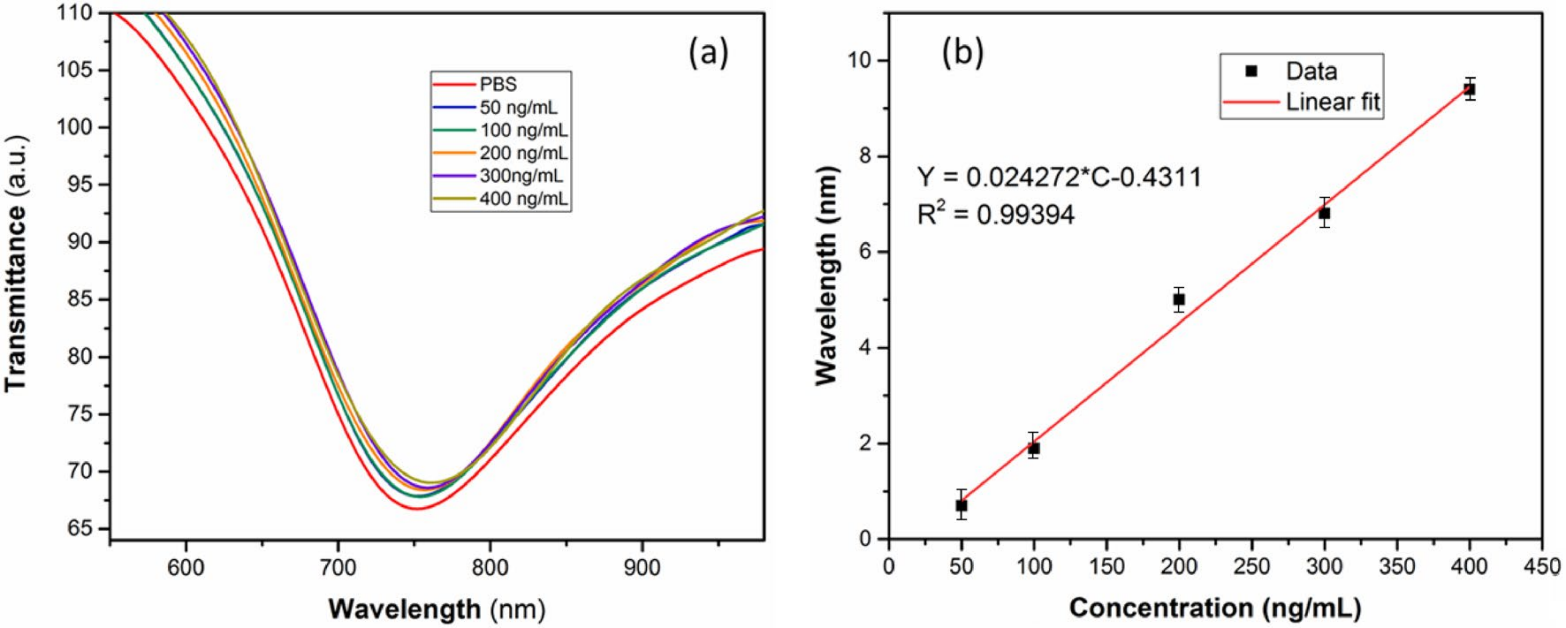
(**a**): SPR wavelength shifts of the Ab(Ft)/L-Cysteine/MoS_2_/OF-SPR biosensor with respect to different concentrations to Ferritin; (**b**): Corresponding linear calibration curve, all measurements are an average of 3 readings.

Figure 6a and b show the response of the Ab(Ft)/L-Cysteine/MoS_2_/OF-SPR biosensor and the corresponding calibration curve for varying concentrations (50 to 400 ng/mL) of Ferritin. A red shift in the SPR wavelength is attained in proportion to the increasing concentration of Ferritin. The calibration curve indicates a linear relationship between the SPR wavelength shift and Ferritin concentrations.

Based on the slope analysis of the calibration curve, the sensitivity of the Ab(Ft)/L-Cysteine/MoS_2_/OF-SPR biosensor for Ferritin is estimated to be 0.024 nm per ng/mL. The limit of detection (LOD) has been calculated by the following formula:

LOD = 3σ/sensitivity, where σ stands for the standard deviation of the sensor toward a blank (PBS) solution (i.e., 0.10).

The LOD of the Ab(Ft)/L-Cysteine/MoS_2_/OF-SPR biosensor for Ferritin is found to be 12 ng/mL, which satisfies the clinical requirements. Further, the reproducibility and stability of the sensor response has also been checked. The sensors were prepared in different batches, and it was observed that the responses were stable within ± 5% limit.

The binding affinity of Ferritin towards the Ab(Ft)/L-Cysteine/MoS_2_ has been evaluated by fitting the calibration curves to the Langmuir isotherm model, expressed as below:

1 $$\partial_{SPR} = {{\left[ {\partial \theta_{\max } \times C} \right]} \mathord{\left/ {\vphantom {{\left[ {\partial \theta_{\max } \times C} \right]} {\left[ {\left( {1/k} \right) + C} \right]}}} \right. \kern-0pt} {\left[ {\left( {1/k} \right) + C} \right]}}$$

where *∂θ*_*max*_ is the maximum SPR shift, *C* refers to the concentration of the analyte, and *K* is the affinity constant. Based on the analysis, the value of binding affinity (*k*) between Ferritin and Ab(Ft)/L-Cysteine/MoS_2_ surface is estimated to be 1.17 × 10^6^ M^−1^.

**Figure 7:**
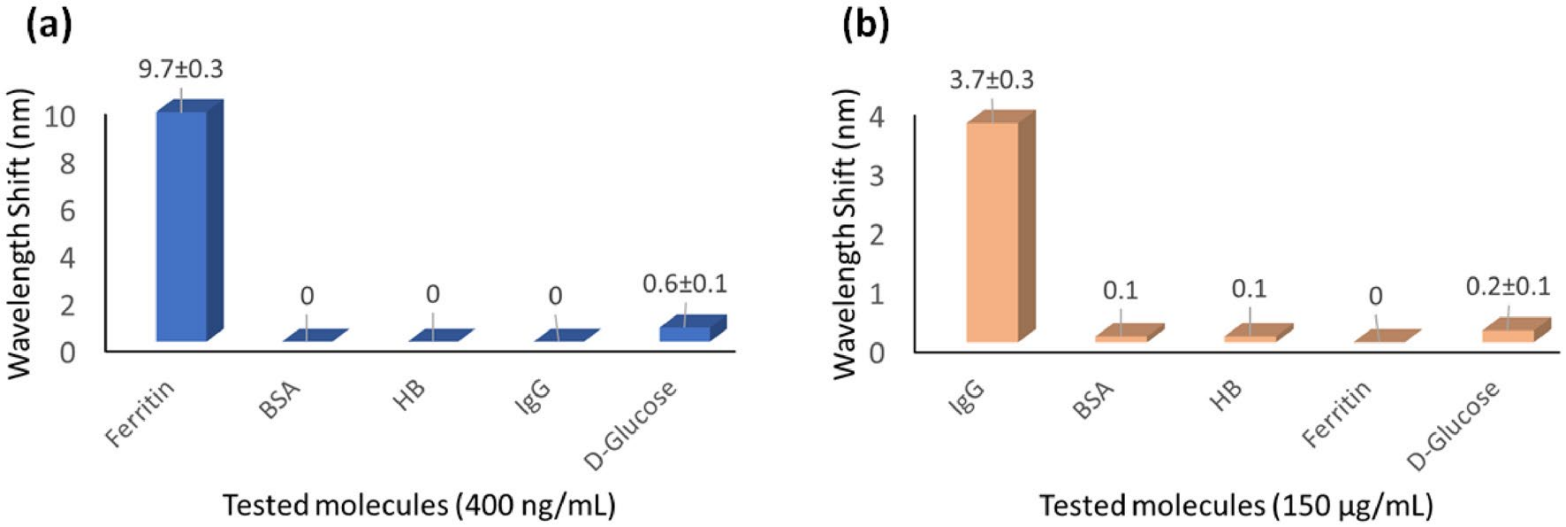
(**a**): Specificity analysis of Ab(Ft)/L-Cysteine/MoS_2_/OF-SPR biosensor; (**b**): Specificity analysis of Ab(IgG)/L-Cysteine/MoS_2_/OF-SPR biosensor. Wavelength shifts are plotted with respect to Ferritin (**a**)/IgG (**b**) concentration and some other non-specific bioanalytes.

The specificity of the Ab(Ft)/L-Cysteine/MoS_2_/OF-SPR biosensor for Ferritin is investigated with respect to some other possible interfering molecules, e.g., Bovine Serum Albumin (BSA), Hemoglobin (Hb), Human IgG, and D-Glucose. As the results of the study (Fig. 7a) show, the sensor did not produce any noticeable SPR wavelength shift in the presence of non-specific molecules. This speaks about the specific nature of the sensor for Ferritin only.

#### IgG detection

The Ab(IgG)/L-Cysteine/MoS_2_/OF-SPR biosensor has also been designed for the sensing of human IgG. It may be highlighted here that the modification of gold coated optical fiber with L-Cysteine/MoS_2_ is not only useful to immobilize the antibodies via a covalent chemistry but the protective layer of the nanomaterial conjugate over the gold film also allowed us to reuse the same fiber after the Ferritin bioassay experiments. The fiber with L-Cysteine/MoS_2_ and loaded Ferritin antibodies was left in contact with 0.1 M HCl solution for 1 min, followed by repeated washings with deionized water. After cleaning the gold layer, the cleaned fiber was again dipped in a L-Cysteine/MoS_2_ for 30 min for realizing a fresh coating of the nanosheet conjugate. Next, the anti-IgG antibodies were immobilized over the sensor surface as per the steps mentioned previously in the experimental section.

**Figure 8:**
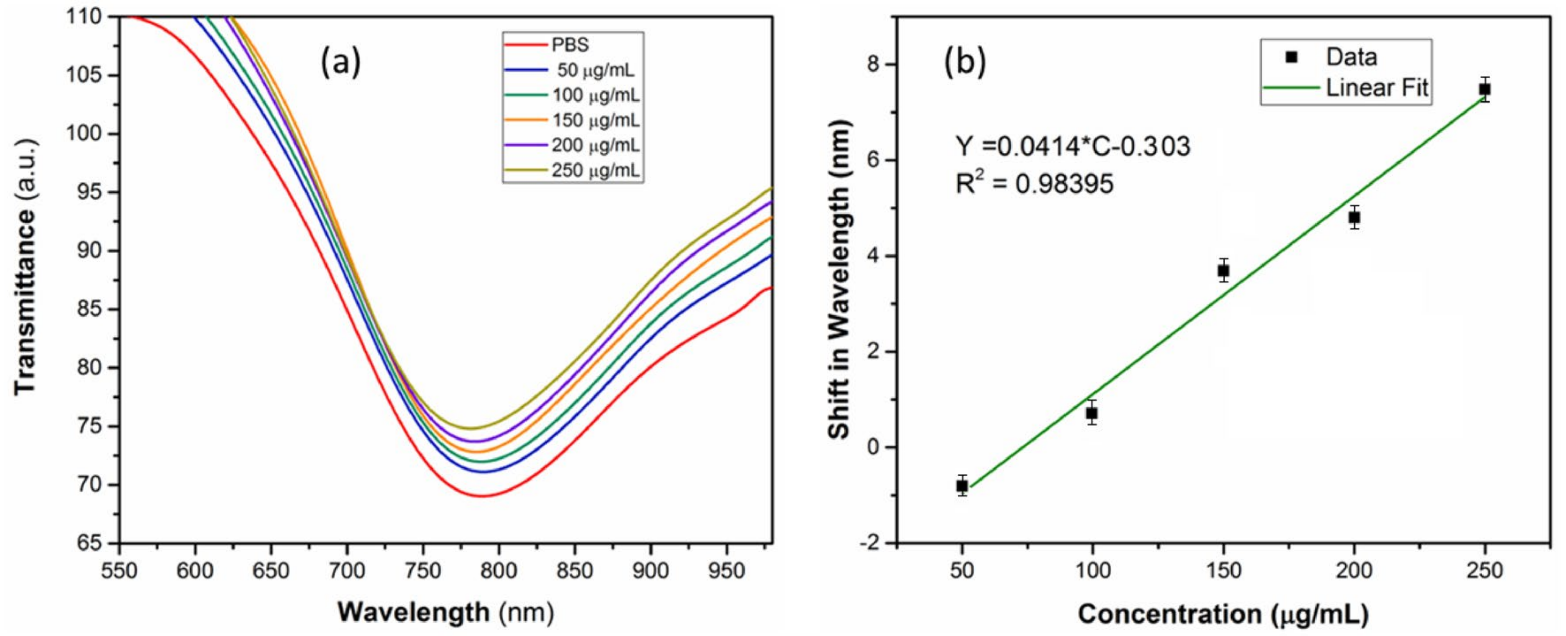
(**a**): SPR wavelength shifts of the Ab(IgG)/L-Cysteine/MoS_2_/OF-SPR biosensor with respect to different concentrations to IgG; (**b**): Corresponding linear calibration curve, all measurements are an average of 3 readings.

The SPR wavelength shifts recorded from the Ab(IgG)/L-Cysteine/MoS_2_/OF-SPR biosensor for 50 to 250 µg/mL of IgG and the corresponding calibration curve are presented in Fig. 8a and b, respectively. For IgG, the sensor has displayed blue SPR wavelength shifts with respect to the increasing concentrations. The nature of shifts, i.e., blue or red, is associated with the refractive index changes, which, in turn, depend upon the range of concentrations being tested. The sensor responses in both cases (Ferritin and IgG) were reproducible in several tests. For IgG, a linear sensor response with a satisfactory extent of regression coefficient is observed within the investigated range of the concentration (Fig. 8b). The sensitivity of the sensor for IgG under the given experimental conditions is assessed to be 0.04 nm per µg/mL. The LOD of the method is determined to be 7.2 µg/mL IgG, which again satisfies the practical requirements.

The reproducibility studies on the IgG detection revealed a response stability within ± 5%. The value of binding affinity (*k*) between IgG and Ab(IgG)/L-Cysteine/MoS_2_ surface is found out to be 5.29 × 10^6^ M^−1^. The specificity of the Ab(IgG)/L-Cysteine/MoS_2_/OF-SPR biosensor for IgG was also studied against Ferritin, BSA, Hb, and D-Glucose (Fig. 7b). Here also, the sensor did not yield any SPR wavelength shift when non-specific molecules were analyzed.

**Figure 9:**
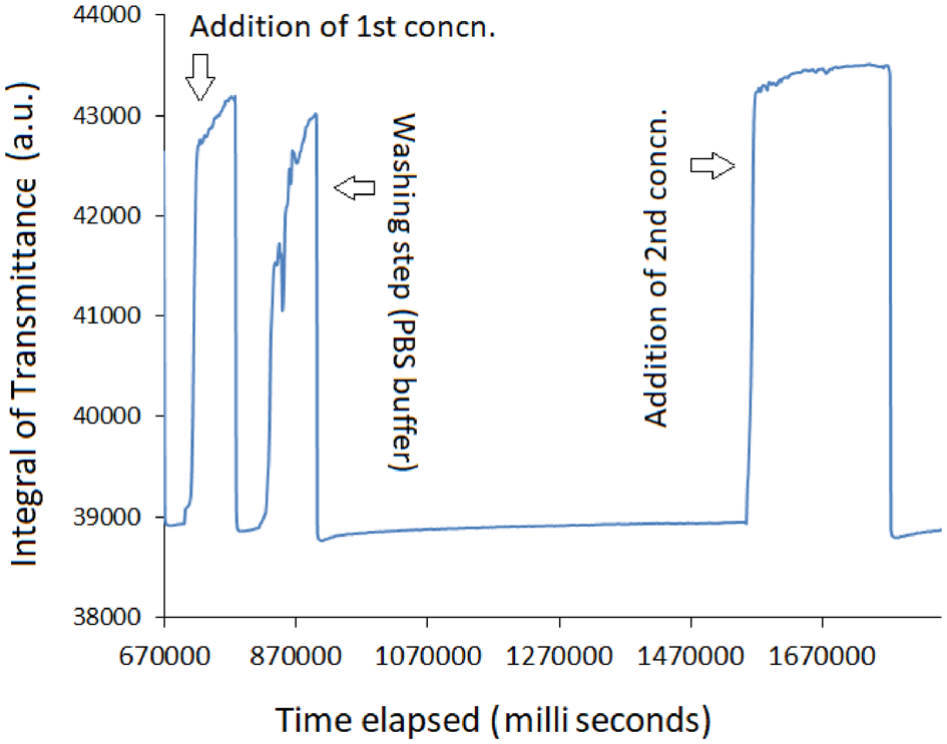
Time dependent transmission response of Ab(IgG)/L-Cysteine/MoS_2_/OF-SPR biosensor for two different concentrations.

As a representative data, the time dependent transmission response of the Ab(IgG)/L-Cysteine/MoS_2_/OF-SPR biosensor for a cycle of two different concentrations is shown in Fig. 9. A simple washing step with PBS buffer in between the two measurements was carried out. The spikes in the washing step represent the flushing of the fiber sensor surface with buffer solution as unattached protein were removed.

A comparison between the performance of the herein reported Ab(Ft)/L-Cysteine/MoS_2_/OF-SPR and Ab(IgG)/L-Cysteine/MoS_2_/OF-SPR biosensors for Ferritin and IgG, respectively with the recently reported similar optical fiber biosensors is summarized in Table 1. Since, to the best of the authors’ knowledge, this is the first work reporting the use of any SPR-OF biosensor for Ferritin, we did not find any competing similar system. For IgG also, the features of the present sensor are of clinical significance.

**Table 1:** Evaluation of the performance of Ab(Ft)/L-Cysteine/MoS_2_/OF-SPR and Ab(IgG)/L-Cysteine/MoS_2_/OF-SPR biosensors with respect to the recently reported similar systems.

Type of optical fiber sensor configuration	Modification layer	Analyte tested and range of detection	Limit of detection	Sensor sensitivity	Ref
L-Cysteine conjugated MoS_2_ nanosheet modified SPR optical fiber	None	Ferritin 50–400 ng/mL	12 ng/mL	0.024 nm/(ng/mL)	This work *(No competing literature)*
		Human IgG 50–250 µg/mL	7.2 µg/mL	0.04 nm/(µg/mL)	This work
H-shaped SPR optical fiber	11-Mercaptoundecanoic acid	Human IgG 10–100 µg/mL	3.4 µg/mL	0.05 nm/(μg/mL)	Huang et al.^42^
D-type SPR optical fiber	Poly dimethyl diallyl ammonium chloride (PDDA) and Poly(sodium-p-styrenesulfonate) (PSS)	Human IgG 0.02–0.08 mg/mL	0.2018 μg/mL	91 nm/(mg/mL)	Chen et al.^29^
Tilted fiber Bragg grating (TFBG) SPR fiber	Graphene oxide (GO) and Staphylococcal protein A	Human IgG 30–100 µg/mL	0.5 µg/mL	0.096 dB/(μg/mL)	Wang et al.^43^
SPR optical fiber	Polydopamine	2–100 µg/mL	2 µg/mL	0.41 nm/(μg/mL)	Shi et al.^44^
Noncore fiber (NCF) sandwiched between two multimode fibers (MMF) SPR sensor	Diallyldimethyl ammonium chloride (PDDA) and styrenesulfonate sodium salt (PSS)	Human IgG 0.025–0.1 mg/mL	1.75 µg/mL	57.06 nm/(mg/mL)	Zheng et al.^28^
U-shaped MoS_2_ nanosheets between gold film and fiber (fiber-MoS_2_-gold film)	Polydopamine (PDA)	Human IgG 5–70 µg/mL	19.7 ng/mL	1.014 nm/(μg/mL)	Song et al.^36^

The regeneration ability of the Ab/L-Cysteine/MoS_2_ modified fiber-optic SPR sensors (both for Ferritin and IgG) is being investigated as a separate study. In some preliminary studies, we have been able to regenerate the sensors by washing them with 10 mM Glycine–HCl buffer (pH 3.0). The regeneration step allowed the reuse of the sensor for at least 2 cycles. However, further detailed studies are continued in this direction to establish the factors like optimum flow rate, volume required of the regeneration buffer, time, number of regenerations without compromising on sensor’s binding affinity, etc.

## Conclusions

The utility of the L-Cysteine conjugated MoS_2_ nanosheets for the preparation of a SPR-OF sensor for Ferritin and IgG are established in this study. The use of L-Cysteine/MoS_2_ conjugate has proven useful to ensure a uniform and stable coating of the nanosheets over the gold layer. It addresses the concerns of peeling-off of the MoS_2_ layers from the optical fiber surface which otherwise are observed when a physical adherence method is applied. This stability of the nanomaterial over the gold film allowed us to carry out several bioassays without the need of repeated nanomaterial coating steps. Furthermore, the adopted strategy also helps in preserving the basic features of the gold coated optical fibers and the same fiber could be used for a number of bioassays. The present study has been undertaken using a low-cost setup (e.g., a portable spectrometer), yet we obtained remarkable sensor sensitivities and LODs for both Ferritin and IgG. The biosensors are suitable for analysis in low as well as high concentration ranges.

## Data Availability

The datasets used and/or analysed during the current study available from the corresponding author on reasonable request.
